# A novel finite element model of the ovine lumbar intervertebral disc with anisotropic hyperelastic material properties

**DOI:** 10.1371/journal.pone.0177088

**Published:** 2017-05-04

**Authors:** Gloria Casaroli, Fabio Galbusera, René Jonas, Benedikt Schlager, Hans-Joachim Wilke, Tomaso Villa

**Affiliations:** 1 Laboratory of Biological Structure Mechanics (LaBS), Department of Chemistry, Materials and Chemical Engineering "Giulio Natta", Politecnico di Milano, Milan, Italy; 2 IRCCS Istituto Ortopedico Galeazzi, Milan, Italy; 3 Institute of Orthopaedic Research and Biomechanics, Center for Musculoskeletal Research (zmfu), Ulm University, Ulm, Germany; University of Pennsylvania, UNITED STATES

## Abstract

The Ovine spine is an accepted model to investigate the biomechanical behaviour of the human lumbar one. Indeed, the use of animal models for *in vitro* studies is necessary to investigate the mechanical behaviour of biological tissue, but needs to be reduced for ethical and social reasons. The aim of this study was to create a finite element model of the lumbar intervertebral disc of the sheep that may help to refine the understanding of parallel *in vitro* experiments and that can be used to predict when mechanical failure occurs. Anisotropic hyperelastic material properties were assigned to the annulus fibrosus and factorial optimization analyses were performed to find out the optimal parameters of the ground substance and of the collagen fibers. For the ground substance of the annulus fibrosus the investigation was based on experimental data taken from the literature, while for the collagen fibers tensile tests on annulus specimens were conducted. Flexibility analysis in flexion-extension, lateral bending and axial rotation were conducted. Different material properties for the anterior, lateral and posterior regions of the annulus were found. The posterior part resulted the stiffest region in compression whereas the anterior one the stiffest region in tension. Since the flexibility outcomes were in a good agreement with the literature data, we considered this model suitable to be used in conjunction with *in vitro* and *in vivo* tests to investigate the mechanical behaviour of the ovine lumbar disc.

## Introduction

Human specimens for *in vitro* experiments on spine biomechanics have been used for decades, despite some intrinsic limitations [1]. Primarily, human specimens are characterized by a strong variability in anatomy and physical properties, and since usually harvested from aged subjects they often present degenerated or pathological features. In addition, the costs of using human specimens are remarkable and often not nimbly sustainable when planning research studies. Therefore, the number of specimens usually collected and assessed in the experiments results reduced, negatively affecting the statistical robustness of the outcomes. Similarly, the use of animal models is characterized by intrinsic disadvantages as genetic, biological, anatomical and postural differences in comparison with humans. Concerning to that, many studies investigated the spinal mobility and failure properties assessing human and animal models, but the use of different species (humans, sheep, calf, pig) [2] caused a lack of comparability between the results [3–8].

However, within the animal substituting options sheep are considered as a favored choice because their disc anatomy shows many similarities with the human one, especially in the lumbar and in the thoracic sections [9]. Sheep spines were used to study the initial stabilizing effect of spinal implants in cervical and lumbar segments [10,11]. Furthermore, the similar histomorphology of the lumbar intervertebral disc (IVD) and good results in fusion techniques made the ovine species also a good model for *in vivo* studies. Moreover, with respect to cows, the housing and maintenance of sheep are cheaper. These peculiarities made sheep a good animal model of the human IVD and promoted the development of experimental tests aimed at exploring the IVD behavior under different loading scenarios.

Despite some investigations about the structure and composition of the ovine IVD are available in the literature [12], to our knowledge only few groups investigated *in vitro* the mechanical properties of the ovine annulus fibrosus. In this regard Kasra and colleagues [13] performed tensile tests on lumbar annular specimens to investigate Young modulus, stress and strain to failure. Little and co-authors [14] performed uniaxial and biaxial compression and shear tests on sheep specimens to investigate the hyperelastic response of the annulus ground substance.

Nonetheless, despite animals can be exploited as a surrogate of humans, there are some economic and ethical reasons for which the number of animals sacrificed for research studies must be reduced. Experiments are expensive, time consuming and laborious. Animal Sacrificing is strictly regulated and the debate on animal use is a daily issue [1]. The European directive 2010/63/EU on the protection of animals used for scientific purposes is based on the principle replacing, reducing and refining the animal experiments [15].

By this point of view, *in silico* models are crucial because they can effectively support experimental studies. Furthermore, they provide additional advantages in comparison with the *in vitro* studies by giving information about local stresses, pressures and deformations, which cannot be directly measured *in vitro*.

Many numerical studies on the IVD were performed, but the majority of them were focused on modeling human segments [16–20]. The combination of numerical analysis and experimental testing can help to better understand the mechanical behavior of the disc, but the anatomical and mechanical differences between the ovine and the human disc [9,21,22] prevent the use of the human models for investigating *in vitro* animal studies. Concerning to that, a finite element model of the ovine IVD that can be used to better understand and predict the mechanical response of the IVD would be very useful.

In view of having a numerical model to analyze the stress state generated by the loading conditions applied in a parallel *in vitro* study [23], the aims of this work are (1) to identify the anisotropic hyperelastic parameters that describe the ovine annulus fibrosus and (2) to obtain a validated biomechanical model of the sheep lumbar IVD. The model would then be used to generate a predictor of the mechanical failure of the ovine IVD [24].

## Materials and methods

### Ethics statement

In this study ovine specimens were collected from animals euthanized with potassium chloride (20 mg/kg IV) involved in another study unrelated to the ongoing research. However, all applicable international, national and/or institutional guidelines for the care and use of animals were followed. Specifically, the Lazzaro Spallanzani Institute Animal Care and Use Committee (IACUC) approved the study designed for this group of animals, in compliance with institutional guidelines as defined in national (Law 116/92, Authorization n.19/2008-A issued March 6, 2008, by the Italian Ministry of Health) and international laws and policies (EEC Council Directive 86/609, OJ L 358. 1, December 12, 1987; Standards for the Care and Use of Laboratory Animals—UCLA, U. S. National Research Council, Statement of Compliance A5023-01, November 6, 1998). The animals were housed at the facilities of the Lazzaro Spallanzani Institute according international standards. A certified veterinarian—responsible for health monitoring, animal welfare supervision, experimental protocols and procedure revision—regularly checked animals. All procedures on animals performed in this study were in accordance with the ethical standards.

### Ground substance characterization

A numerical investigation was performed to identify the optimal combination of material parameters to obtain the most suitable characterization of the ovine lumbar annulus fibrosus. For the calculation of the material properties of ground substance, we replicated the experimental work of Little and colleagues [14] in which the uniaxial compressive tests were performed on sheep lumbar annulus specimens harvested from three different regions of the annulus (anterior, posterior and lateral). A finite element (FE) model of the tested specimens was generated using Abaqus 6.14 (Simulia, Dassaultlut Systèmes, Providence, RI, USA). The model had rectangular shape (3 mm length x 3 mm width x 4 mm height) and the mesh was composed of 4500 elements and 5376 nodes. Linear hexahedral elements were used.

The model was constrained in all degrees of freedom at the nodes of the lower surface. Five displacements of 10%, 15%, 20%, 25% and 30% of the specimen’s height were imposed in the axial direction to simulate uniaxial compression. The stress was calculated as the ratio between the total axial force at the constrained nodes and the transversal area. The isotropic Neo-Hookean material relationship was assigned to the model, and *C*_*10*_ and *D* were changed in a range defined as described below. Since *C*_*10*_ and *D* are related to the equivalent Young modulus E and to the Poisson ratio as

$$C_{10}=\frac{\mu_{0}}{2}$$ (1)

and

$$D=\frac{2}{K_{0}}$$ (2)

where

$$\mu_{0}=\frac{E}{2\left[1+\nu \right]}$$ (3)

and

$$K_{0}=\frac{E}{3\left[1-2\nu \right]}$$ (4)

they were defined assuming E between 0.1 MPa and 3 MPa and keeping the Poisson ν equal to 0.49. A Python script was used to drive the simulations and to read automatically the results for the tested parameter values.

In order to assess the material properties which allow for the best fit with the experimental data, the minimum mean square error with the regression curves provided in the literature paper was calculated distinguishing anterior, posterior and lateral parts. The values associated to the minimum error in the whole full factorial design were assumed for the mechanical description of the ground substance. A linear regression assessment (R^2^) was performed between the experimental and numerical results.

### Characterization of the collagen fibers

The mechanical parameters describing the collagen fiber behavior were obtained by performing traction tests on annulus fibrosus specimens and then using the same full factorial optimization method applied for the ground substance.

**Fig 1:**
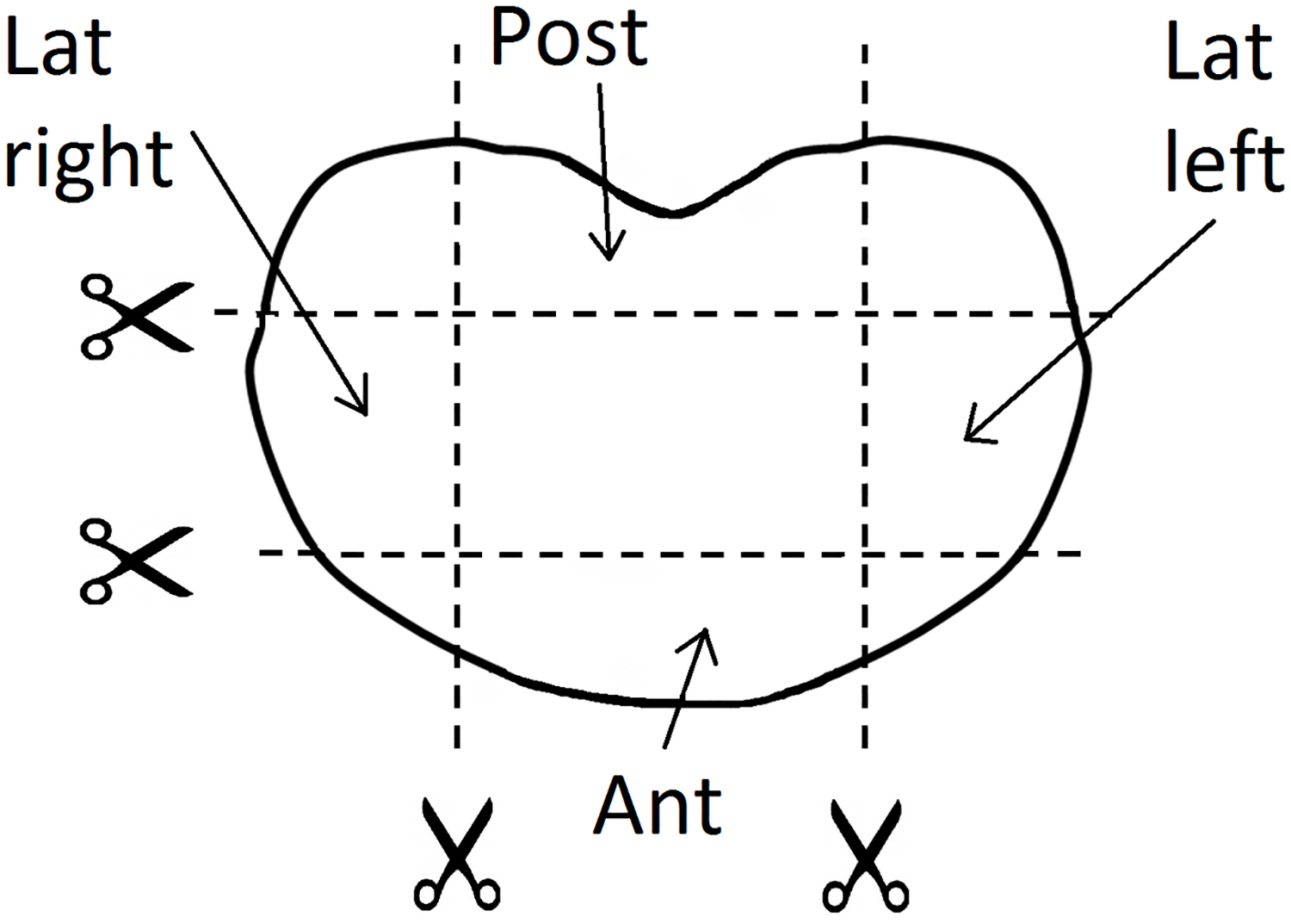
Schematic representation of the preparation of the specimens. The FSUs were cut in four parts corresponding to the anterior (Ant), lateral left (Lat left), lateral right (Lat right) and posterior (Post) annulus. The residues of soft tissues were removed using a scalpel.

**Fig 2:**
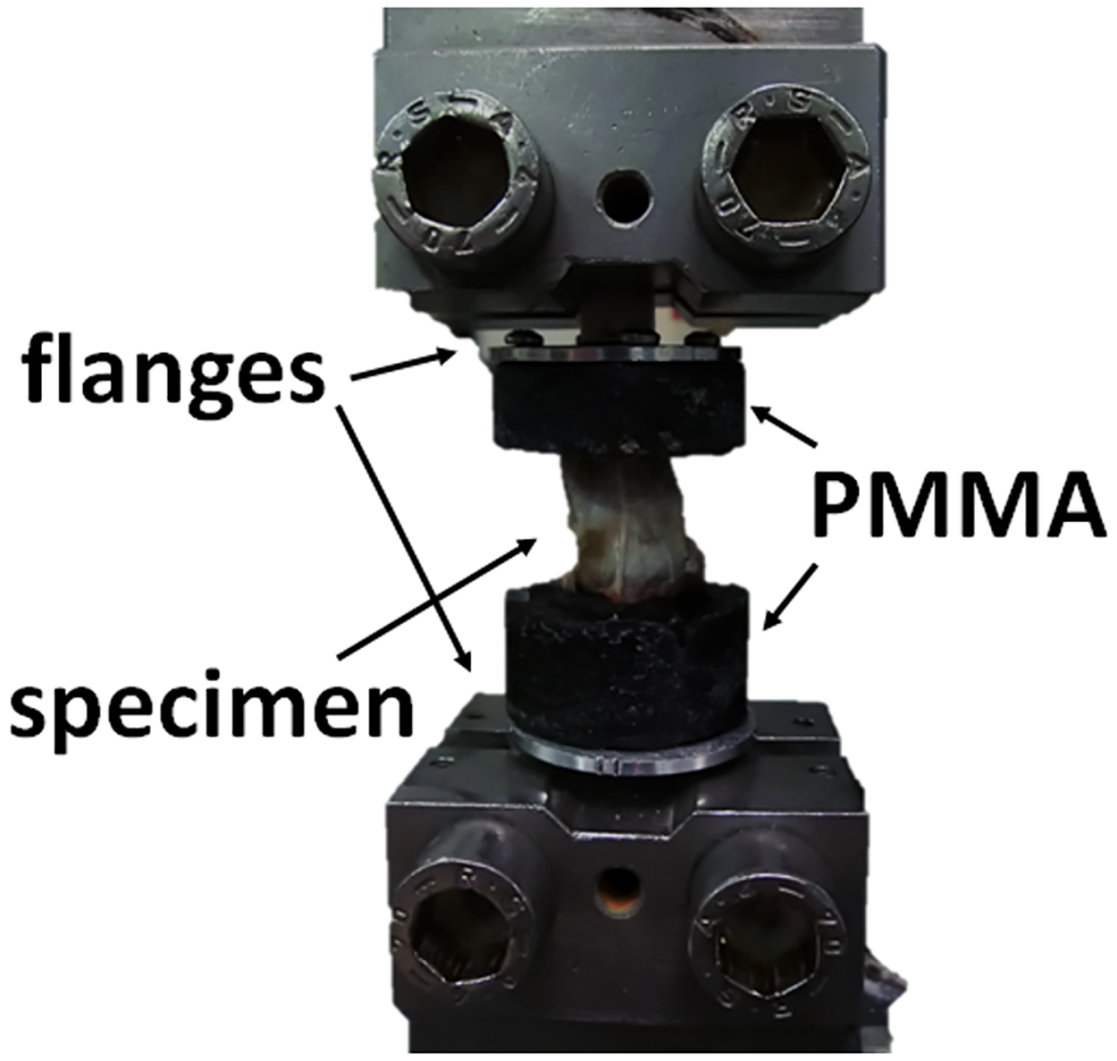
Set-up used for the tensile test. The specimens were fixed in the testing machine using two flanges screwed into the PMMA. The specimens were blocked at the bony extremities and stretched to failure.

Twenty specimens of annulus were extracted from 4 sheep lumbar spines stored at -20°C (mean age 4 years). The spines were collected from animals euthanized with potassium chloride (20 mg/kg IV) involved in another study unrelated to the ongoing research [25]. The spines were cut in the middle of each vertebra in the transversal plane by means of a saw, obtaining 5 Functional Spinal Units (FSUs) per spine. Subsequently, annulus specimens were obtained by cutting each FSU in the frontal and in the sagittal plane (Fig 1) saving the bone extremities. The specimens were divided in 3 groups (anterior n = 8, lateral n = 6 and posterior n = 6). In order to ensure the independence of the results of each annulus location on the IVD level, specimens from the entire lumbar spine were included in each group. The height of each specimen was measured using a caliper. The median height was 4 mm (range 3–5.6), 4.5 mm (range 3.3–4.6) and 2.3 mm (range 2–3) in the anterior, lateral and posterior regions respectively. Iron wires (diameter 1.5 mm) were inserted in the bone to ensure the fixation in the subsequent embedding in PMMA (Technovit 6091, Heraeus Kulzer, Wehrheim, Germany). The specimens were again stored at -20°C. Before testing, the specimens were thawed in salin solution for 12 hours; flanges were screwed onto the PMMA and the specimens were placed in the testing machine (Synergie 200H, MTS System Corporation) (Fig 2). For preconditioning of the specimens, 4 cycles up to 5% of strain at the speed of 0.01 mm/sec were applied, then the specimens were brought to failure at same strain rate. After testing, each specimen was cut at half the height using a scalpel and pictures were taken with a standarized photographic set-up. The pictures were loaded in Mimics 17.1 (Materialise HQ, Leuven, Belgium) and the cross-sectional area of each specimen in the transverse plane was manually measured. The median area was 142 mm^2^ (range 98–178), 125 mm^2^ (range 71–203) and 118 mm^2^ (range 101–141) in the anterior, lateral and posterior parts respectively. The stress—strain curves were calculated. For the material characterization within the physiological limits, the median stress values between specimens of each group corresponding to strain lower than 0.6 were calculated.

**Fig 3:**
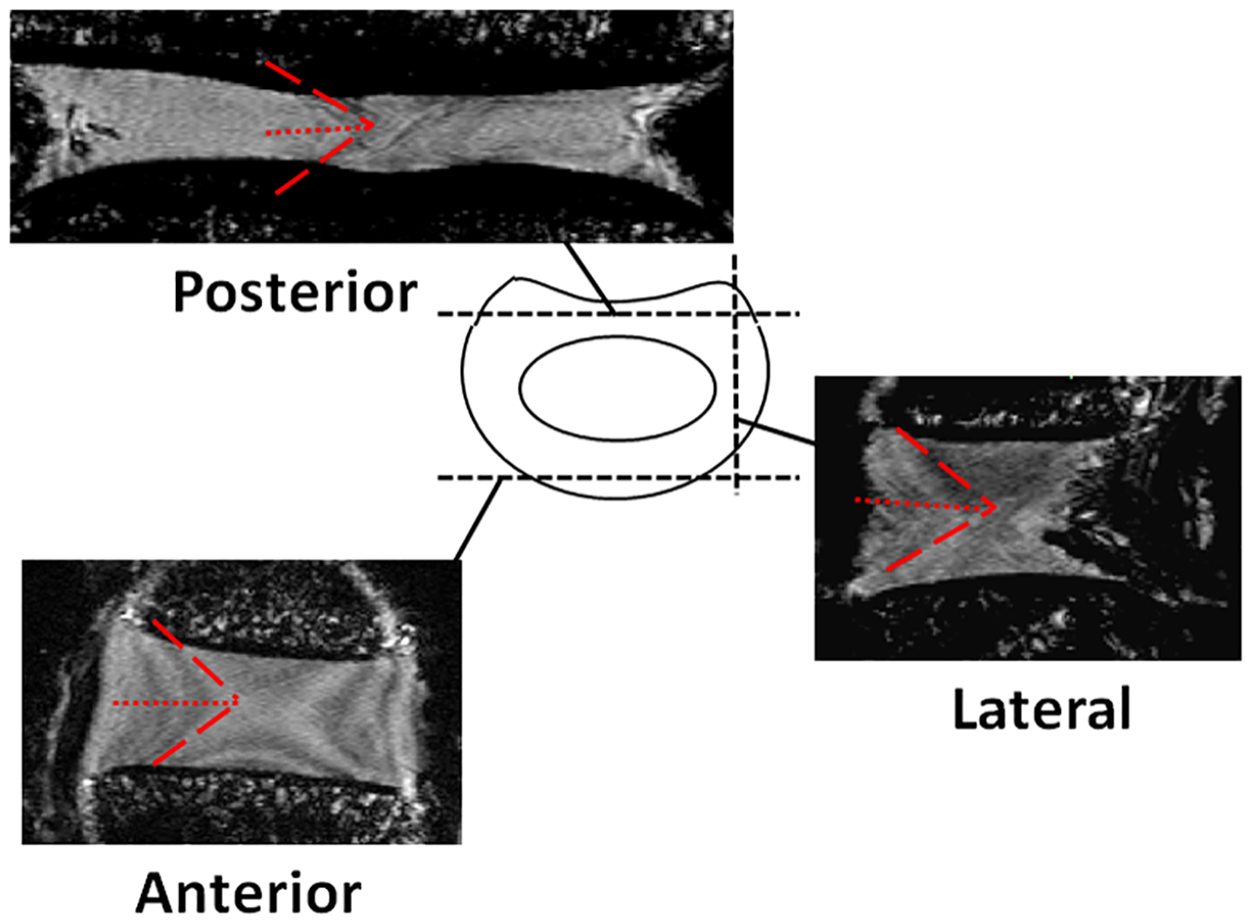
Schematic representation of the method used for the measurement of the collagen fiber orientation. Three sections (anterior, lateral and posterior) for each set of MR images of six IVD were captured and the angles between the fibers were manually measured. Half of the angles measured were considered as the orientation respect to the horizontal plane.

For the measurement of the fiber orientation, Magnetic Resonance (MR) scans of six lumbar IVDs were taken from another study [22]. The specimens were representative of the whole lumbar spine (1 X L1-2, 1 X L2-3, 1 X L3-4, 2 X L4-5, 1 X L5-6) and they all belonged to different animals. The specimens were scanned with a dedicated 11.7 T small animal MRI system (BioSpec 117/16, Bruker Biospin, Ettlingen, Germany; MR-method: FLASH, contrast: T1, Resolution: 100 μm isotropic, acquisition time: 15 min). MR images were processed with Mimics 17.1 to measure the angle between fibers in frontal and sagittal planes for anterior, posterior and lateral annulus fibrosus by four different observers. After having assessed that the fibers measurements had a Gaussian distribution and that under Chebyshev's inequality a minimum of 95% of values lied within two standard deviations, the average values of each region were calculated (Fig 3).

Subsequently, a FE model of the specimens of annulus tissue was generated. Due to the shape differences among the specimens, based on the hypothesis that the stress-strain curve of the specimen is independent on the physical dimensions, we created a model with a simple rectangular geometry and dimensions similar to those of the specimens (15 mm length x 8 mm width x 4 mm height). The mesh of the model was composed by 7600 elements and 9009 nodes, linear hexahedral elements were used. The annulus fibrosus was described using the Holzapfel-Gasser-Ogden (H-G-O) anisotropic hyperelastic formulation [26,27].

The formulation considers an exponential material behavior for the fibers and a non-linear Neo-Hookean behavior for the ground matrix.

Therefore, the strain energy potential is composed by the contributes of both the matrix and the fibers:

$$U=C_{10}\left(\bar{I_{1}}-3\right)+\frac{1}{D}\left(\frac{{\left(J^{el}\right)}^{2}-1}{2}-\;\text{ln}J^{el}\right)+\;\frac{k_{1}}{2k_{2}}\sum_{\alpha =1}^{N}\left\{exp\left[k_{2}{\langle {\bar{E}}_{\alpha }\rangle }^{2}\right]-1\right\}$$ (5)

where ${\bar{E}}_{\alpha }$ is a strain-like quantity that characterizes the deformation of the fibers and is defined as:

$${\bar{E}}_{\alpha }\;\underline{\underline{\text{def}}}\;\kappa \left(\bar{I_{1}}-3\right)+\left(1-3\kappa \right)\left[\bar{I_{4\left(\alpha \alpha \right)}}-1\right]$$ (6)

*C*_*10*_ and *D* describe the matrix compressibility and the matrix stiffness, and correspond to the Neo-Hookean material parameters. *k*_*1*_ and *k*_*2*_ describe the stiffness and the non-linearity of the fibers, respectively, whereas and *κ* indicates the fiber dispersion. The formulation assumes that all the fibers have the same mechanical properties and the same dispersion: *κ* = 0 means that the fibers are perfectly aligned, *κ* = 1/3 defines the maximum degree of dispersion and the material becomes isotropic. $\bar{I_{1}}$ is the first deviatoric strain invariant, *J*^*el*^ is the elastic volume ratio and $\bar{I_{4\left(\alpha \alpha \right)}}$ are the pseudo-invariants of the distortion part of the right Cauchy-Green strain and unit vectors in the fiber directions. The H-G-O form assumes that the collagen fibers can supports tension only, because they would buckle under compressive loading: the mathematical consequence is that the anisotropic contribution in the strain energy function appears only when ${\bar{E}}_{\alpha }\;>\;0$, that means a positive strain of the fibers (Abaqus Documentation 6.14, 2014. Anisotropic Hyperelastic behavior, 22.5.3).

We assumed *κ* = 0.01 which corresponds to a minimal fiber dispersion. As for the ground substance parameter research, we defined a range for *k*_*1*_ and *k*_*2*_ according to the values found in the literature [26,27] and we used a Python script to obtain the values that ensure the best-fit with the experimental curves through a full factorial design (n = 3115 simulations).

The model was constrained as the one described previously, and six displacements (10%, 20%, 30%, 40%, 50% and 60% of the height of the model) in the axial direction were imposed to simulate a traction test. For all simulations, we calculated the stress as the ratio between the total force in axial direction at the constrained surface and the area, and the mean square error with the polynominal curves were calculated. *k*_*1*_ and *k*_*2*_ values associated to the minimal errors were subsequently used to model the entire IVD.

### Lumbar IVD modeling and validation

**Fig 4:**
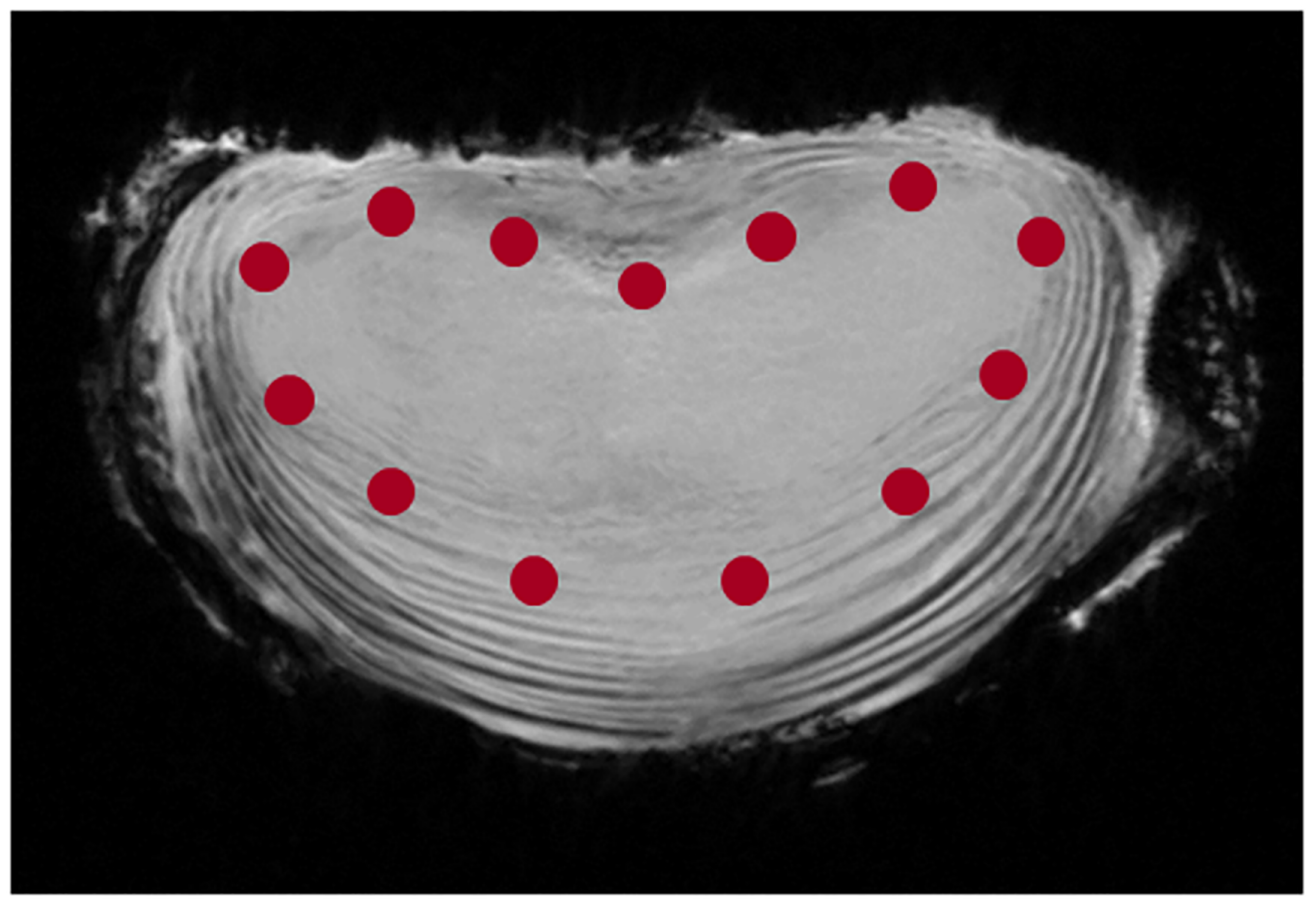
Schematic representation of the method used for the distinction of the nucleus pulposus and the annulus fibrosus. A set of landmarks was defined to distinguish the disc structures for the generation of the IVD model. The structures were distinguished using a global windows method based on the gray values.

**Fig 5:**
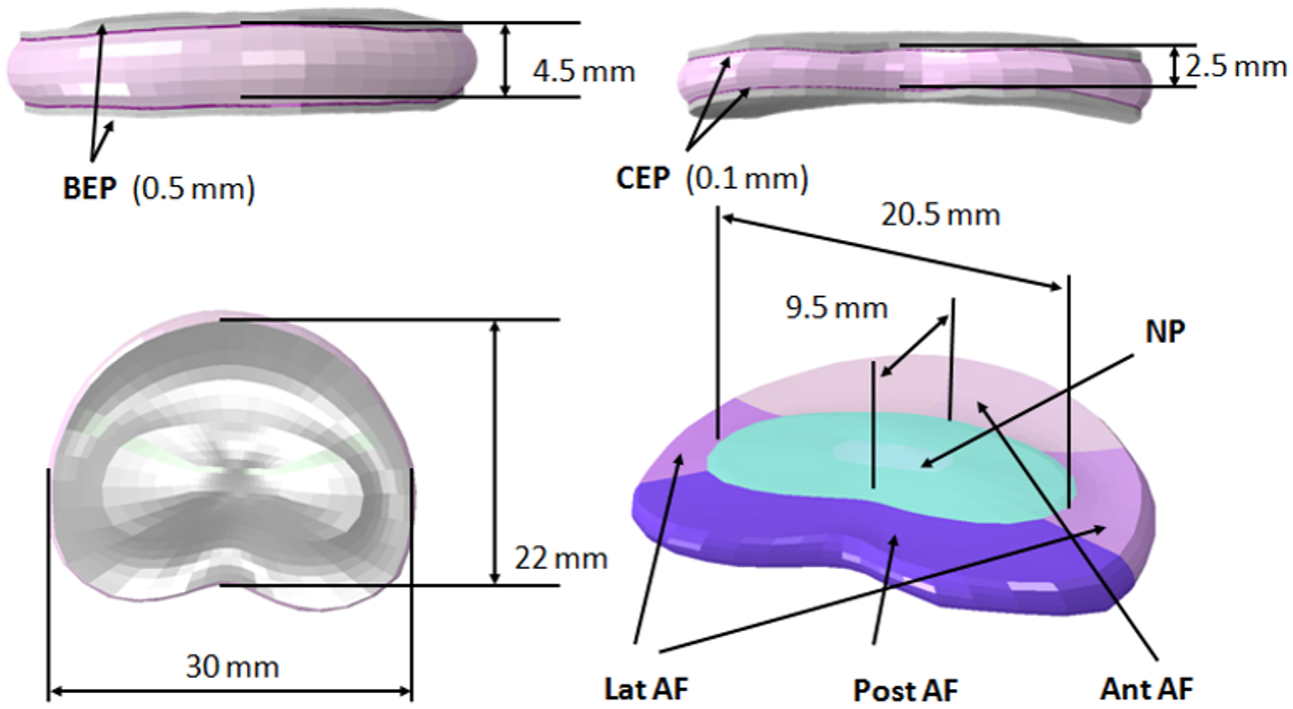
Geometry and composition of the FE model. The IVD model was composed by the nucleus pulposus (NP), the caudal and cranial cartilagineous endplates (CEPs) and bony endplates (BEPs) and the annulus fibrosus (AF). The AF was divided in anterior (Ant AF), lateral (Lat AF) and posterior (Post AF) parts.

A FE model of the lumbar IVD was generated. A micro-computed tomography (μ-CT) (Skyscan 1172, Skyscan, Kontich, Belgium) of a L3-4 frozen FSU was performed at 34 μm resolution using a voltage of 100 kV and a current of 100 μA with an acquisition time of 20 minutes, and the caudal and cranial surface of the vertebrae endplates were reconstructed using segmentation software (Avizo 8.0, FEI, Oregon, USA). The geometries were used for the construction of the FE model using a custom Python script. The regions of the annulus fibrosus and the nucleus pulposus were defined by using a set of landmarks which were originally taken from MRI data of the same segment (Fig 4). The raw mesh of the FE model of the disc was then divided into the following sub-sets: nucleus pulposus, the anterior, lateral and posterior annulus, the cartilaginous endplates and the bony endplates (Fig 5). The model had 5137 linear hexahedral elements and 5994 nodes. The H-G-O constitutive relationship was assigned to the annulus fibrosus; two families of fibers were defined and the orientation previously measured was assigned to the anterior, lateral and posterior regions. *C*_*10*_, *D*, *k*_*1*_ and *k*_*2*_ values found with previously described method were imposed. Material properties of the endplates and of the nucleus pulposus were taken from the literature (Table 1) [28].

**Table 1:** Material properties assigned to the ovine disc model.

	*C*_*10*_ (MPa)	*D*_*1*_ (MPa^-1^)	*k*_*1*_ (MPa)	*k*_*2*_	*κ*	Fibers orientation
**Ant AF**	0.061	0.311	24	1700	0.01	±29°
**Lat AF**	0.033	0.615	5	940	0.01	±30°
**Post AF**	0.077	0.261	1	50	0.01	±28°
**NP**	0.168	0.12				
	**E (MPa)**	**ʋ**				
**CEP**	24	0.4				
**BEP**	1000	0.3				

Ant AF, Anterior Annulus Fibrosus; Lat AF, Lateral Annulus Fibrosus; Post AF, Posterior Annulus Fibrosus; CEP, Cartilagineous Endplate; BEP, Bony Endplate.

The model was constrained in all directions at all nodes of the inferior surface of the caudal endplate. Intradiscal pressure in the unloaded condition was set to 0.2 MPa [29] using an UMAT subroutine, thus generating a pre-tensioning that simulated the mechanical effect of disc swelling. The mechanical range of motion (ROM) of the disc model was calculated after applying pure moments of 3.75 Nm in axial rotation, flexion-extension and lateral bending. For the sake of validation the results were then compared with *in vitro* data [29].

## Sensitivity analysis

A sensitivity study of the most influent tissue model parameters on the local stresses and the global disc stiffness was performed. E was changed between 0.1 and 1 MPa, *k*_*1*_ between 1 and 45 MPa whereas *k*_*2*_ between 1 and 2000. Four values were chosen for each parameter, hence 64 combinations of material parameters were investigated. A Python script was used to obtain the different combinations of material parameters and to drive the simulations in a full factorial design. The ranges of motion in flexion-extension, right lateral bending and right axial rotation were calculated. A section in the postero-lateral region of the annulus fibrosus was defined, according to the region of the annulus where failure experimentally occurred under complex loading conditions [24]. The average maximum principal stress in the circumferential and in the axial directions was calculated. The influence of each H-G-O parameter on the disc flexibility and on the average stress was invetigated and the results were reported using box-plots.

## Results

### Ground substance characterization

**Fig 6:**
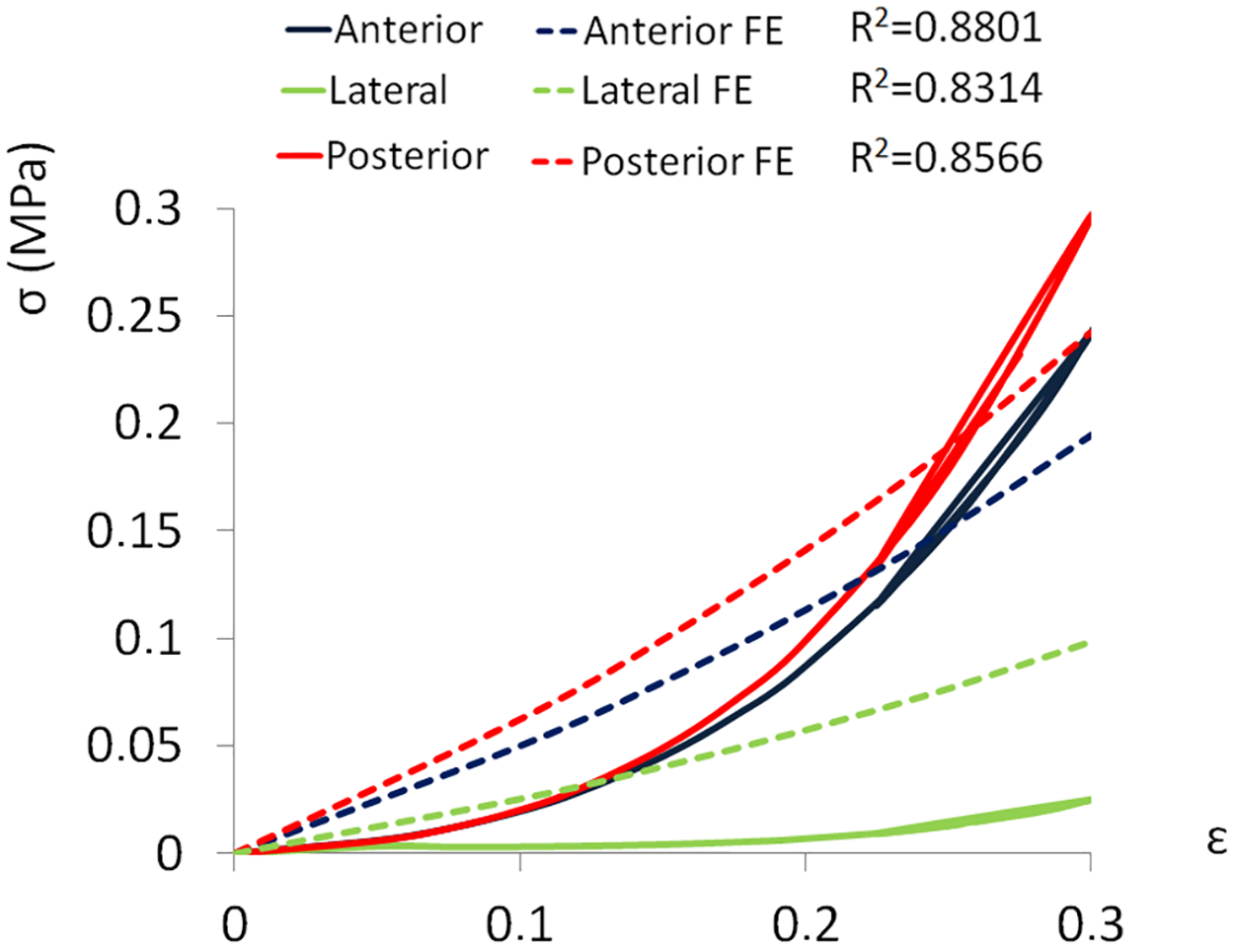
Mechanical response to compression of the ground substance. Numerical and experimental stress—strain curves of the ground substance in all annulus regions. The curves represent the best fit with the experimental data. The linear correlation coefficient (R^2^) between the experimental and numerical stresses related to the same strain are reported.

The ground substance resulted stiffer in the posterior annulus (E = 0.46 MPa), followed by the anterior (E = 0.38 MPa) and the lateral one (E = 0.26 MPa). A linear regression assessment between the numerical and experimental results [14] showed a R^2^ higher than 0.8 (Fig 6). As a matter of fact, the assumed Neo-Hookean material formulation was not capable of fully capturing the non-linear nature of the experimental findings.

### Fibers characterization

**Fig 7:**
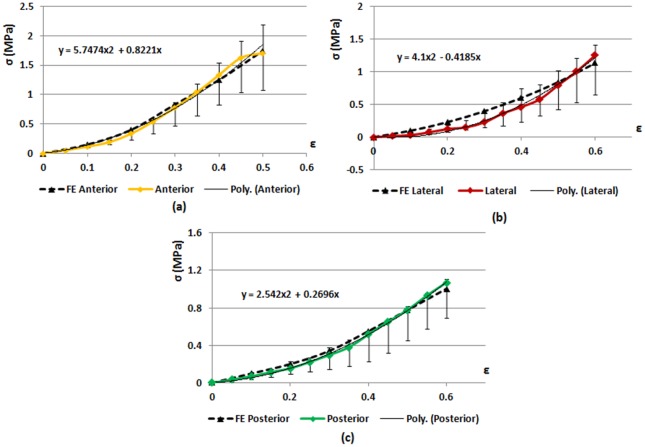
Comparison between finite element simulations and experimental tests of the uniaxial tension response of the annulus fibrosus. Experimental and numerical stress—strain curves of the (a) anterior, (b) lateral and (c) posterior specimens. Anterior, Lateral and Posterior represent the experimental results; FE Anterior, FE Lateral and FE Posterior represent the numerical results; Poly. is the quadratic polynomial interpolation of the experimental results. The experimental curves represent the median values of the stresses. For each measured value, the first and the third quartile are reported. Median values were used to calculate stress-strain response because of the non-normality of the distribution.

The measurements of the fiber orientation had a normal distribution and under Chebyshev’s inequality, a minimum of 95% of values lied within two standard deviations. The average values of the fiber angles were ±29° (SD = 3.9°), ±30° (SD = 4.0°), and ±28° (SD = 4.1°) in the anterior, lateral and posterior regions of the annulus, respectively. The average yield stresses were 2.85 MPa (SD = 0.79), 1.95 MPa (SD = 0.86) and 2.06 MPa (SD = 0.26) for anterior, lateral and posterior parts respectively, corresponding to strains of 0.5, 0.6 and 0.6 (Fig 7). The Young modulus was calculated as the tangent stiffness in the axial direction and resulted 5.6 MPa (SD = 1.4), 2.6 MPa (SD = 1.2) and 2.7 MPa (SD = 0.6) in the anterior, lateral and posterior regions respectively.

Distinct properties were found for anterior, lateral and posterior regions (Table 1). The anterior part had the highest stiffness, followed by the lateral and the posterior ones. *k*_*2*_ was up to 900 in the anterior and lateral regions while it was lowest in the posterior region.

### IVD model validation

**Fig 8:**
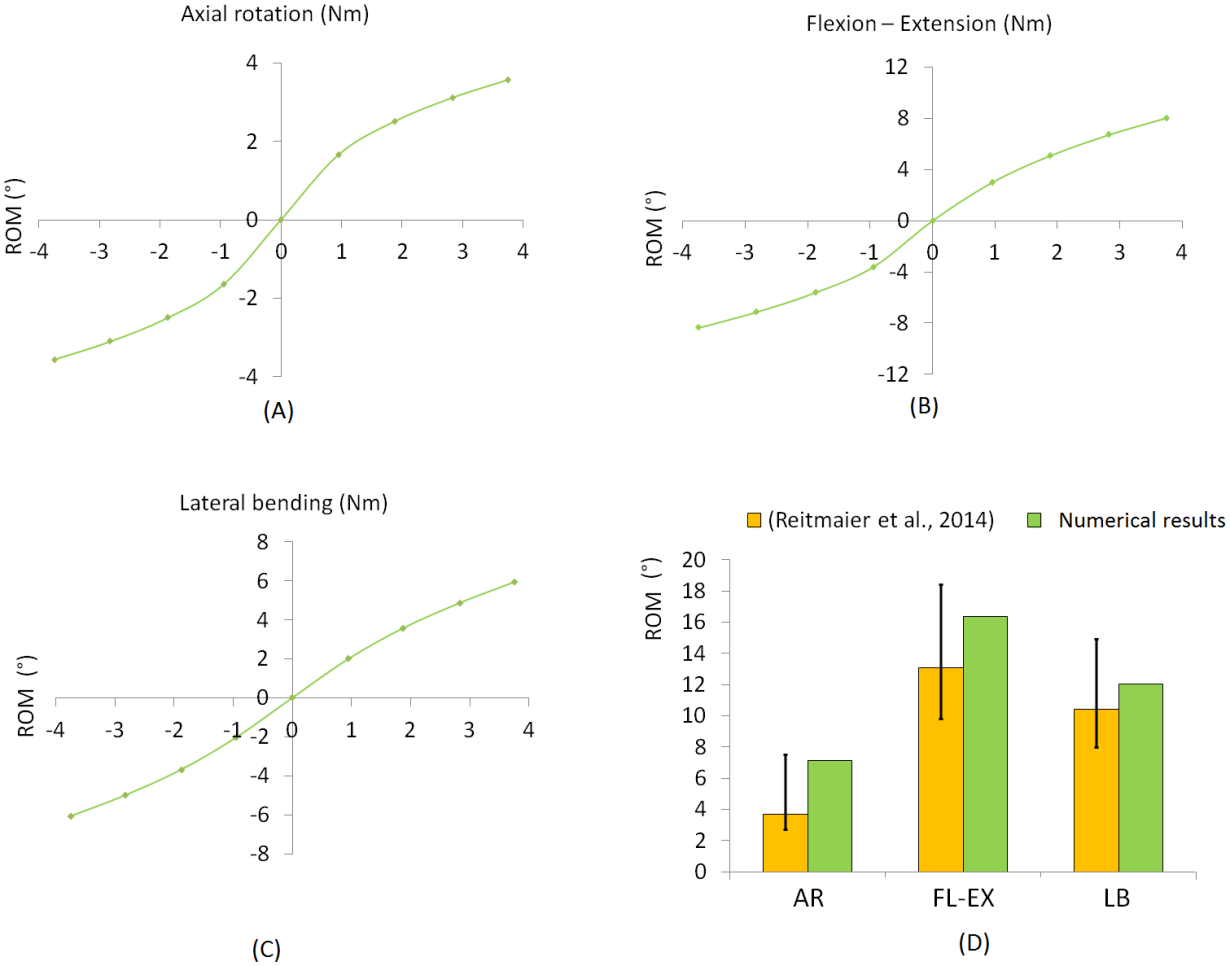
ROM curves and comparison with *in vitro* data. Mechanical response of the numerical model to (A) axial rotation (AR), (B) flexion-extension (FE), and (C) lateral bending (LB). For each loading scenario a maximum moment of 3.75 Nm was applied to the cranial BEP to replicate *in vitro* tests. Histograms in (D) compare the ROM calculated in the finite element simulations with the literature.

In FE simulations conducted on the entire IVD, ROMs were 7.1°, 16.4° and 12° for axial rotation, flexion-extension and lateral bending respectively, showing a good fit with experimental data [30], with a tendency towards a higher flexibility in all directions (Fig 8).

### Sensitivity analysis

**Fig 9:**
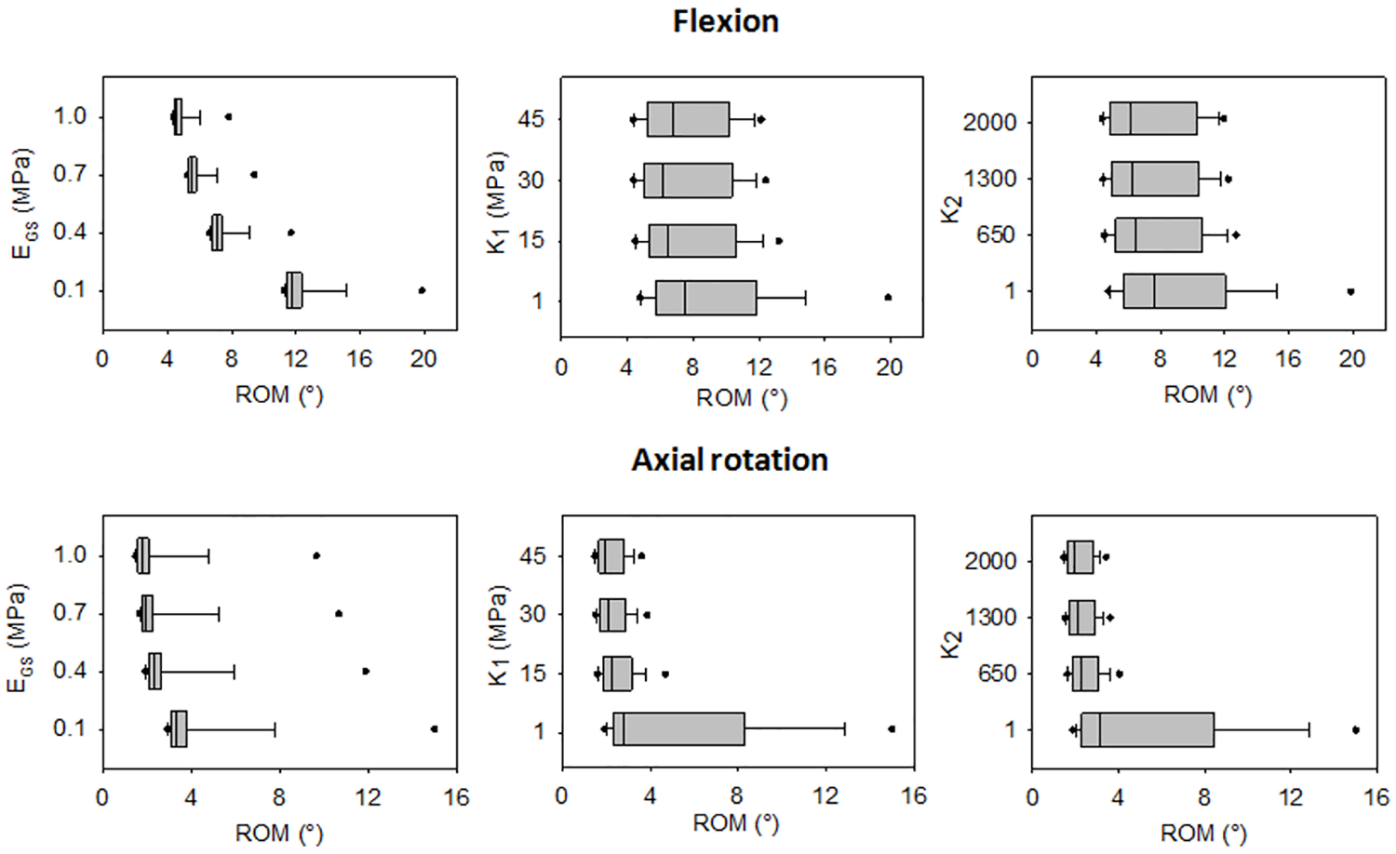
Box-plots of the ranges of motion obtained in flexion and in axial rotation. The ranges of motion (ROMs) of the disc model were calculated for each combination of parameters under the application of a pure moment of 3.75 Nm. E_GS_ is the Young modulus of the ground substance. For each assigned value, the distribution of the corresponding ROM is reported.

The sensitivity analysis revealed a strong influence of the ground substance parameters on both the disc flexibility and the principal stresses. Keeping *C*_*10*_ and *D*_*1*_ constant, the flexibility of the model under physiological rotations showed a low variability (Fig 9 and S1 Fig). On contrast, a large range of flexibility was obtained by the combination of a specific value of *k*_*1*_ or *k*_*2*_ with the other H-G-O parameters, except for axial rotation (Fig 9). In general, increasing *C*_*10*_, *D*_*1*_, *k*_*1*_ and *k*_*2*_ caused a decrease of the ranges of motion.

**Fig 10:**
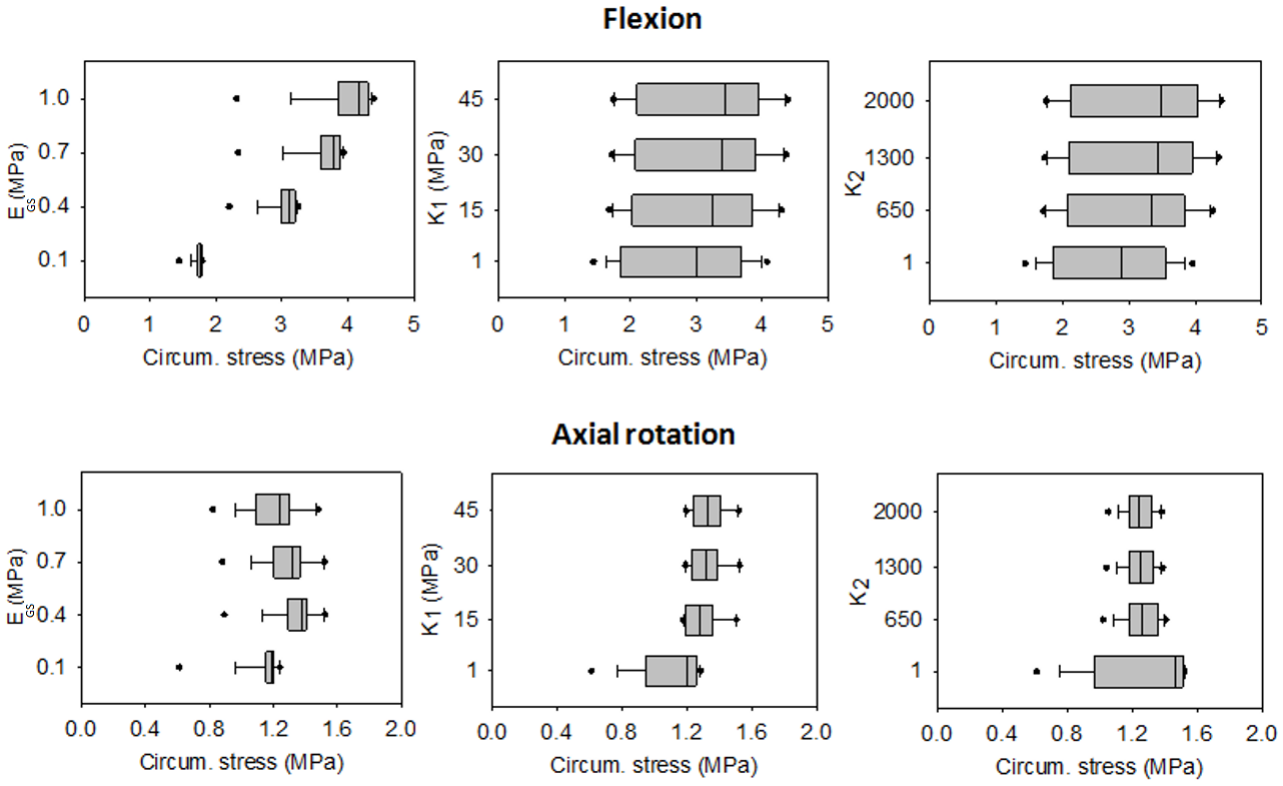
Box-plots of the circumferential stresses obtained by the flexion and the axial rotation. The circumferential (circum.) stresses were calculated for each combination of parameters under the application of a pure moment of 3.75 Nm. E_GS_ is the Young modulus of the ground substance. For each assigned value, the distribution of the corresponding stresses is reported.

The ground substance parameters had a stronger influence on the principal stresses than *k*_*1*_ and *k*_*2*_ (Fig 10). Whereas in flexion and in extension the circumferential and the axial stresses showed a large variability of the results depending on the value assigned to each parameter (S2 and S3 Figs), the difference between the maximum and the minimum values in lateral bending and axial rotation were lower than 1 MPa (S2 and S3 Figs).

## Discussion

In this study, a novel FE model of the ovine lumbar IVD was developed. A FE optimization analysis based on literature data and on purposely conducted *in vitro* tests was performed to investigate the mechanical properties of the annulus fibrosus. The model was subsequently validated by comparing the predicted flexibility in flexion-extension, lateral bending and axial rotation with the literature data [30].

For the ground substance we used data presented by Little and co-authors [14] in which the characterization of the matrix alone was conducted due to the absence of collagen fibers coupling the two cartilaginous endplates of the specimens. Little and colleagues described the ground substance as ‘reinforced-ground matrix’ that was refer to the mechanical function of the ground substance with fibers embedded, but not actively bearing axial load. Since these experimental results were therefore not influenced by the collagen fibers component, we exploited them to characterize the mechanical behavior of the ground substance. However, the validity of the hypothesis was not quantified because of the experimental limitations of isolating the ground substance. This assumption together with the property of isotropy, may explain the lack of agreement between the experimental (clearly non-linear) and the numerical (mostly linear) results. The H-G-O constitutive law includes a contribution of the ground substance, which is described by the same parameters (*C*_*10*_ and *D*_*1*_) used in the Neo-Hookean formulation of isotropic hyperelastic solids. We therefore fitted the properties of the ground substance using the latter material constitutive law, and subsequently adding the contribution of the fibers by means of fitting tensile tests.

In the experimental study, Little and co-authors performed uniaxial compression test up to strain 0.5–0.6, but in the present work the test was limited to 0.3, thus limiting the applicability of the results only to the physiological loading. Predicted Young modulus values were 0.38 MPa, 0.26 MPa and 0.46 MPa in the anterior, lateral and posterior annulus respectively, in a reasonable agreement with the results of Freeman et al [31], who performed dynamic mechanical analyses on human annulus specimens and found a storage modulus in the range of 0.1–0.35 MPa. Although R^2^ values between experimental and numerical results were higher than 0.8, the assumed Neo-Hookean formulation was not capable of fully capturing the non-linear nature of the experimental findings. This disagreement may be due to the fact that the lamellae and the secondary fibers were not included in the models of the ground substance. However, the simulations of the tensile tests which were described using the H-G-O formulation showed a good agreement between the numerical and the experimental results (Fig 6), and the numerical outcomes were mostly within the range defined by the first and the third quartile.

The investigation of the annulus fibers stiffness was conducted by performing tensile tests on annulus specimens distinguishing the anterior, the lateral and the posterior parts, since it has been demonstrated that the mechanical properties differ in the various areas of the annulus as the collagen content [12–14]. The range of mechanical properties of the annulus fibers found in the literature is large and the values are not always comparable with the present study, due to differences in the testing protocols and specimens preparation. Many studies investigated the elastic modulus for the single lamella [32,33], others measured the elastic modulus in different orientations (longitudinal, radial or circumferential orientation) [34–37]. In these studies the Young modulus resulted in a range between 0.1 and 100 MPa. In the current study, the Young modulus of the multilamellar specimens resulted higher in the anterior AF than in the lateral and in the posterior regions, and it was in the range defined in the literature. The range of values for *k*_*1*_ was fixed between 1 and 45 MPa and range for *k*_*2*_ was assumed between 1 and 2000. *k*_*1*_ resulted highest in the anterior annulus and lowest in the lateral and posterior annulus while *k*_*2*_ was higher anteriorly and laterally than posteriorly. The stiffness parameters of the H-G-O formulation found in humans were similar to our results: Malandrino and colleagues found *k*_*1*_ of 2 and 5 MPa in the anterior and in the posterior regions, respectively [17]; Vena and co-authors fixed *k*_*1*_ to 3 MPa in all the regions [27].

The sensitivity of the model to the H-G-O parameters was investigated. It has been demonstrated that under physiological rotations the ground substance has a strong influence on both the disc flexibility and on the stress generated in the annulus. *C*_*10*_ and *D*_*1*_ related to a Young modulus of 0.1 MPa, and *k*_*1*_ and *k*_*2*_ values of 1 caused an excessive flexibility of the model. However, for the sake of simplicity, in this analysis the H-G-O parameters were kept equal in the anterior, lateral and posterior regions. This limitation avoided investigating the effect of the different mechanical properties among the regions of the annulus fibrosus. The median average stress measured in the postero-lateral annulus was higher in the circumferential than in the axial direction. This could be due to the fact that the application of physiological conditions maintains the horizontal component of the collagen fibers orientation higher than the vertical one. In fact, when an axial rotation is applied and the collagen fibers are more involved, the values assigned to *k*_*1*_ and *k*_*2*_ are more influent than for the other rotations (Fig 10). On contrast, when high loads are applied the axial deformation of the annulus is prevalent, with the generation of stresses higher in the axial direction than in the circumferential one [24].

Regarding the measurement of the collagen fibers orientation, since the thickness of the scans was 0.1 mm and the one of the ovine lamellae ranges between 0.1 and 0.5 mm [37] we hypothesized that the resolution of the images was high enough to capture the fiber orientation.

Reid and colleagues [12] showed that in sheep lumbar IVDs fiber angle is 28° in the inner anterior annulus, 30° in the anterior outer annulus and 23° in the posterior annulus. We found a value of ±29°, ±30° and ±28° in the anterior, lateral and posterior parts respectively, which are in a good agreement with the values found in the literature [12]. The normal distribution of the fibers measurements and Chebyshev's inequality test allowed assuming that the collagen fibers within each region of the annulus were close to the mean value. Regards to humans, several measurement-based descriptions of human AF fibre angles have been reported in the literature [38]: Cassidy and co-authors reported that the interlamellar angle decreased from 62 to 45 degrees from the edge of the disc inward to the nucleus [39]. Holzapfel and colleagues reported that the fiber angle is smallest at the midsagittal ventral position (23°) and increases linearly with circumferential position up to the double value (47°) at the midsagittal dorsal position. Regarding this specific aspect, the ovine IVD appears not to be an optimal model of the human IVD.

Other groups published a FE model of the ovine IVD: Schmidt and Reitmaier [21] generated a poroelastic model that completely uses mechanical parameters tailored to the sheep species. The annulus was modeled as a Neo-Hookean material and the collagen fibers were represented by rebar elements. Furthermore, the authors distinguished the inner and outer annulus but maintained the same material properties in the anterior, lateral and posterior parts, in contrast to the present study. Similarly, the fiber orientation was not adjusted to the ovine disc, but was taken from measurements on human specimens. Although the literature model was in good agreement with *in vitro* tests, the use of a continuum model as the one presented in this study instead of a rebar-reinforced material may reduce the computational cost of simulations and may provide an alternative approach for the simulation of complex loading situations. Reutlinger and co-authors [40] proposed a model of the sheep lumbar IVD with anisotropic hyperelastic properties of the annulus fibrosus: they assigned to the ground matrix Neo-Hookean parameters derived through a compression test not explained by the authors. We simulated the experimental tests of Little and colleagues with the parameters suggested, and found that the response of the material was not in good agreement with the experimental data. In contrast with our study, even though the model of Reutlinger and colleagues [40] included refined features such as fiber-matrix and fiber-fiber interactions, the insufficient validation against *in vitro* tests determined an unrealistic response requiring further developments.

Despite the general agreement between the predicted ROMs and *in vitro* results, there are some limitations to highlight. For the ground substance characterization, the presence of the collagen fibers was neglected. Nevertheless, in the experimental test the presence of the collagen fiber gave a contribution to the material resistance because of the interactions with the ground substance. Mechanical tests on annulus specimens also presented some limitations that could have influenced the results. The biological samples presented a remarkable variability in shape and dimension, depending by which lumbar segment and annulus part they were from. Furthermore, we hypothesized that the cross sectional area under tensile load was equal to the area in the unloaded condition, thus leading to an underestimation of the stresses. Besides, no sensitivity analysis on the disc mesh was performed.

Despite of this, the model is in a good agreement with the flexibility values present in the literature. Thus, it is reasonable to adopt the current model to investigate the mechanical behavior of the intervertebral disc in conjunction with or as an alternative of experimental tests, in order to reduce and partly replace them.

## Supporting information

S1 Fig Box-plots of the ranges of motion obtained in lateral bending and extension. The ranges of motion (ROMs) of the disc model were calculated for each combination of parameters under the application of a pure moment of 3.75 Nm. E_GS_ is the Young modulus of the ground substance. For each assigned value, the distribution of the corresponding ROM is reported.
(TIFF)

S2 Fig Box-plots of the circumferential and of the axial stresses obtained by the flexion. The circumferential (circum.) and the axial stresses were calculated for each combination of parameters under the application of a pure moment of 3.75 Nm. E_GS_ is the Young modulus of the ground substance. For each assigned value, the distribution of the corresponding stresses is reported.
(TIF)

S3 Fig Box-plots of the circumferential and of the axial stresses obtained by the extension. The circumferential (circum.) and the axial stresses were calculated for each combination of parameters under the application of a pure moment of 3.75 Nm. E_GS_ is the Young modulus of the ground substance. For each assigned value, the distribution of the corresponding stresses is reported.
(TIF)

S4 Fig Box-plots of the circumferential and of the axial stresses obtained by the lateral bending. The circumferential (circum.) and the axial stresses were calculated for each combination of parameters under the application of a pure moment of 3.75 Nm. E_GS_ is the Young modulus of the ground substance. For each assigned value, the distribution of the corresponding stresses is reported.
(TIF)

S5 Fig Box-plots of the circumferential and of the axial stresses obtained by the axial rotation. The circumferential (circum.) and the axial stresses were calculated for each combination of parameters under the application of a pure moment of 3.75 Nm. E_GS_ is the Young modulus of the ground substance. For each assigned value, the distribution of the corresponding stresses is reported.
(TIF)

S1 File Numerical and experimental data used for the generation of the numerical model. The excel file contains the data on which the numerical model of the intervertebral disc has been generated and validated. ‘Little data’ sheet: experimental results of the compressive tests of the ground substance specimens [14]. ‘Ground Substance’ sheet: numerical data of the finite element models of the ground substance in the anterior, later and posterior region. ‘Anterior’, ‘Lateral’, ‘Posterior’ sheets: experimental results of the traction tests of the annulus specimens in the anterior, lateral and posterior region, respectively. ‘Analysis’ sheet: elaboration of the experimental and of the numerical results of the tensile tests of the annulus fibrosus numerical models. ‘ROM’ sheet: numerical results of the range of motion of the final element model of the intervertebral disc.
(XLSX)
