# Supplementary figures and images for: A novel finite element model of the ovine lumbar intervertebral disc with anisotropic hyperelastic material properties
Source: PLoS One. 2017 May 4;12(5):e0177088. doi: 10.1371/journal.pone.0177088 (PMC5417645; doi:10.1371/journal.pone.0177088)

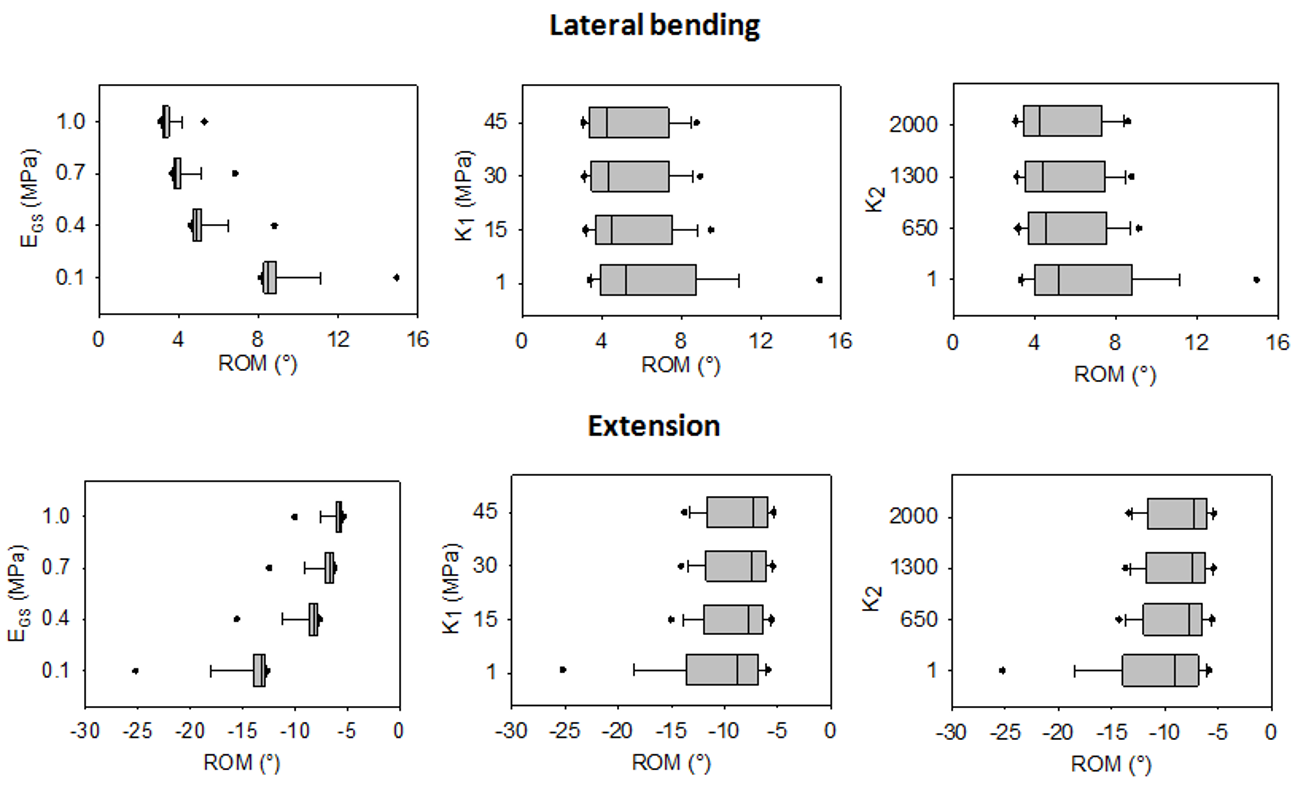

Supplement: S1 Fig — The ranges of motion (ROMs) of the disc model were calculated for each combination of parameters under the application of a pure moment of 3.75 Nm. EGS is the Young modulus of the ground substance. For each assigned value, the distribution of the corresponding ROM is reported. (TIFF) [file pone.0177088.s001.tiff]

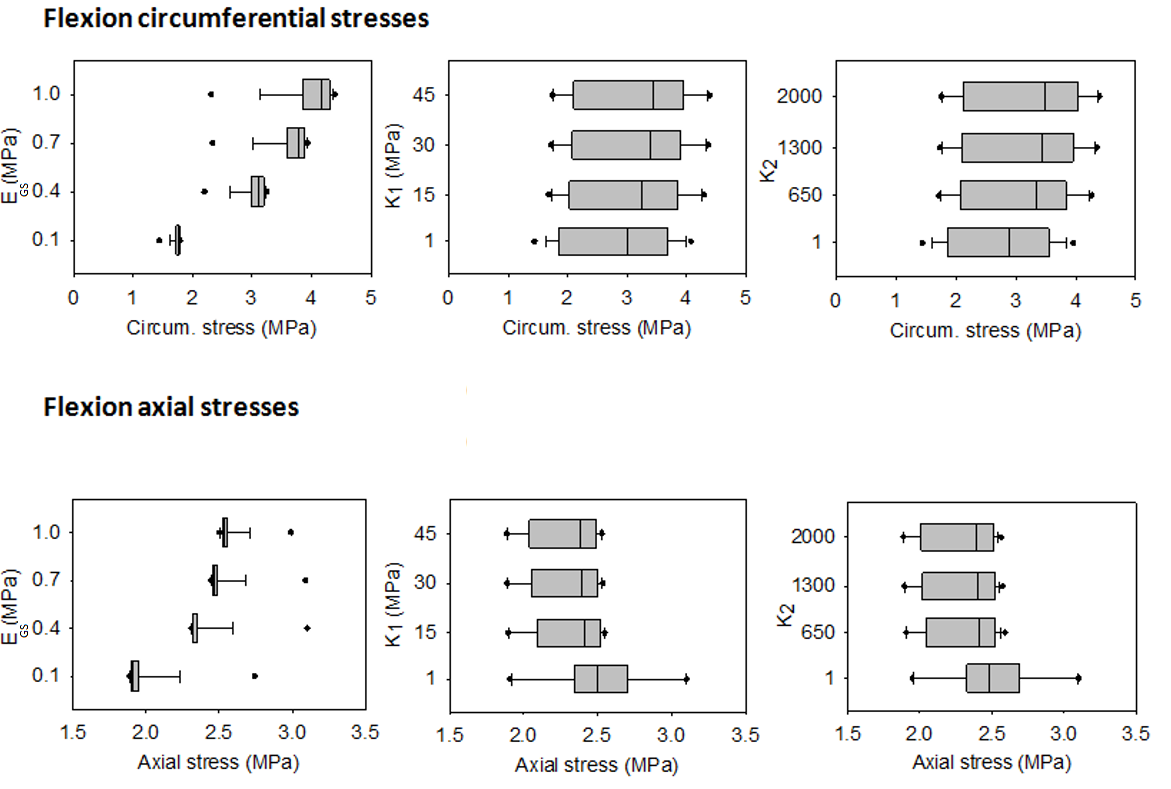

Supplement: S2 Fig — The circumferential (circum.) and the axial stresses were calculated for each combination of parameters under the application of a pure moment of 3.75 Nm. EGS is the Young modulus of the ground substance. For each assigned value, the distribution of the corresponding stresses is reported. (TIF) [file pone.0177088.s002.tif]

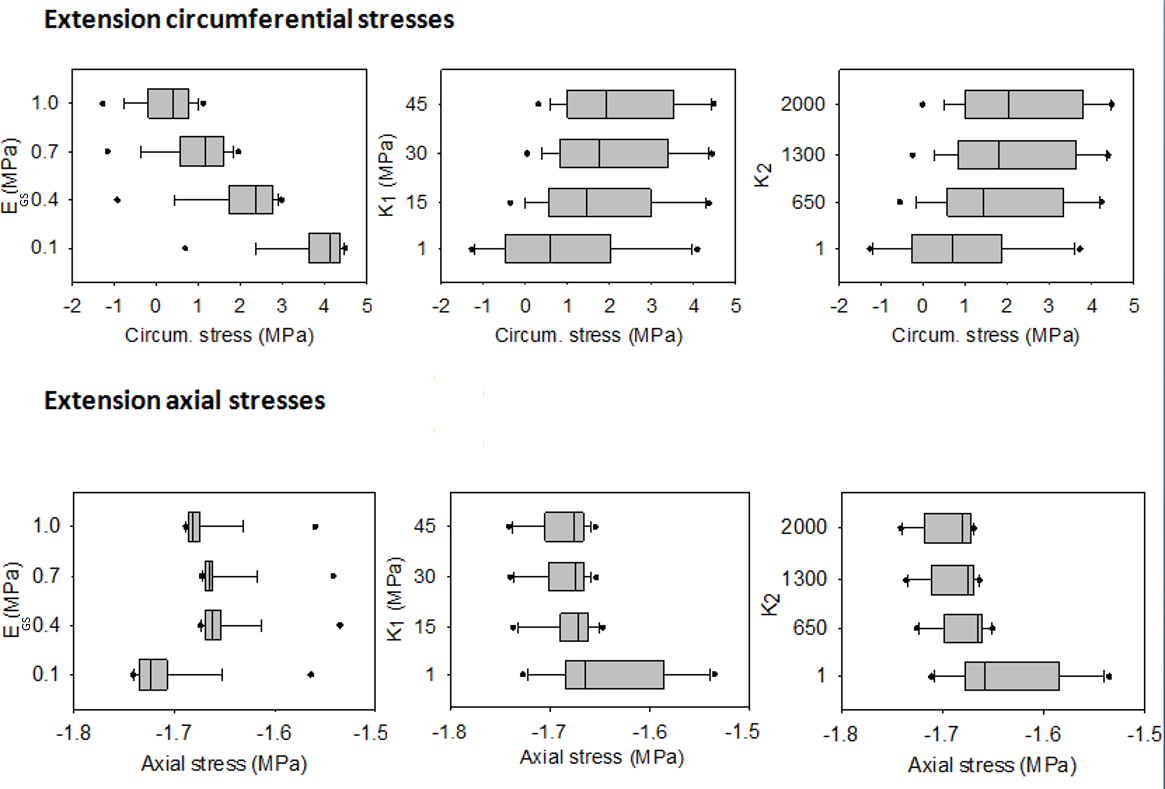

Supplement: S3 Fig — The circumferential (circum.) and the axial stresses were calculated for each combination of parameters under the application of a pure moment of 3.75 Nm. EGS is the Young modulus of the ground substance. For each assigned value, the distribution of the corresponding stresses is reported. (TIF) [file pone.0177088.s003.tif]

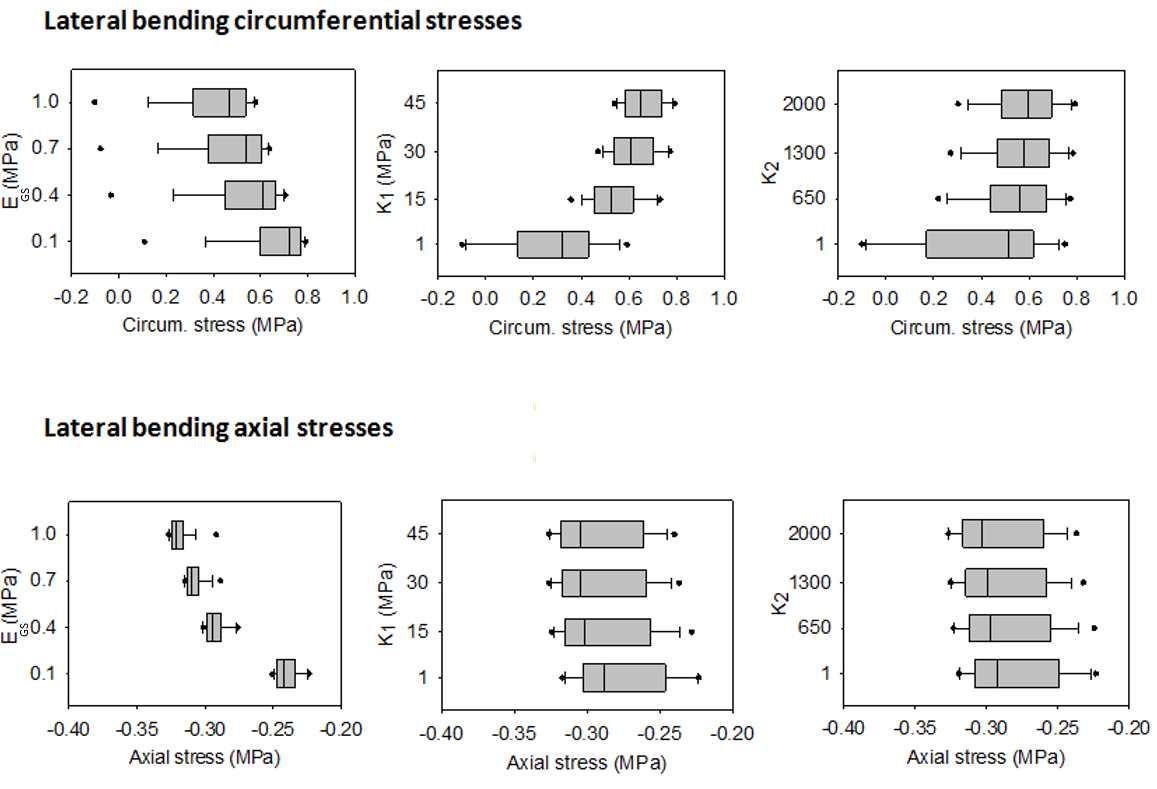

Supplement: S4 Fig — The circumferential (circum.) and the axial stresses were calculated for each combination of parameters under the application of a pure moment of 3.75 Nm. EGS is the Young modulus of the ground substance. For each assigned value, the distribution of the corresponding stresses is reported. (TIF) [file pone.0177088.s004.tif]

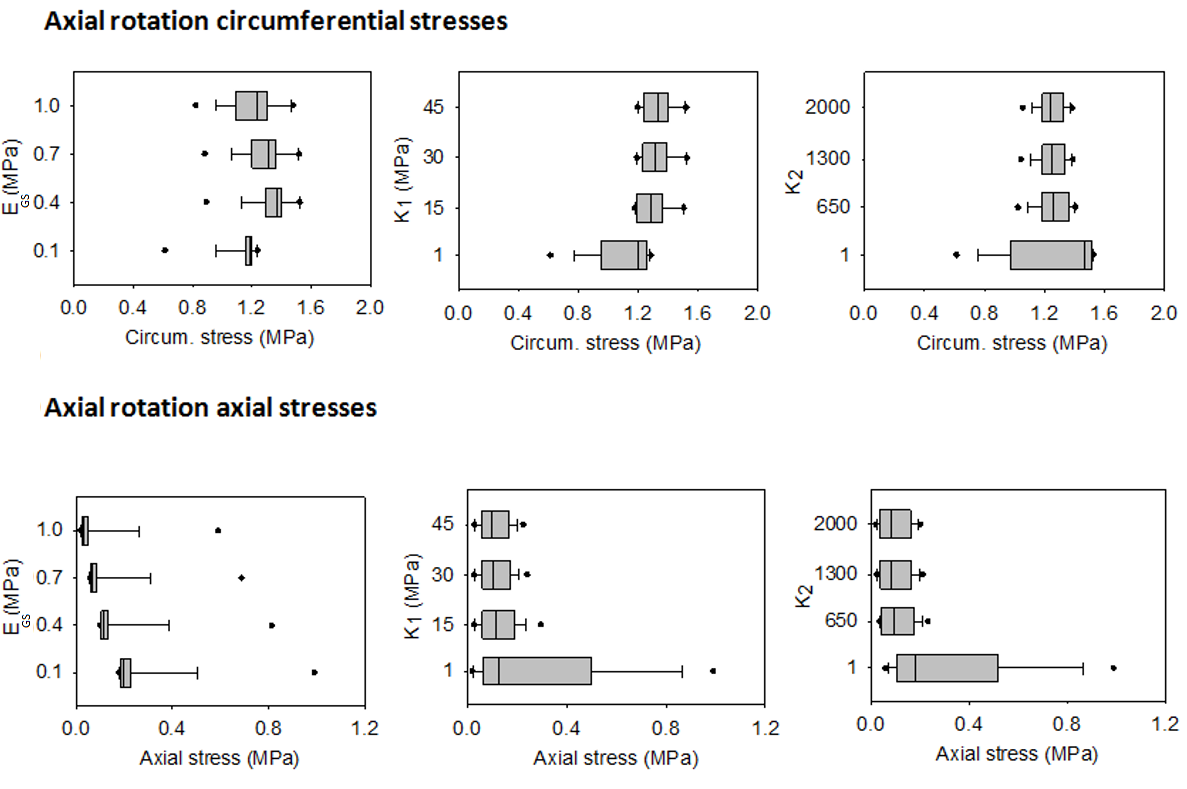

Supplement: S5 Fig — The circumferential (circum.) and the axial stresses were calculated for each combination of parameters under the application of a pure moment of 3.75 Nm. EGS is the Young modulus of the ground substance. For each assigned value, the distribution of the corresponding stresses is reported. (TIF) [file pone.0177088.s005.tif]
